# Ubiquitin specific peptidase 5 enhances STAT3 signaling and promotes migration and invasion in Pancreatic Cancer

**DOI:** 10.7150/jca.48536

**Published:** 2020-09-30

**Authors:** Jie Lian, Chao Liu, Xin Guan, Bojun Wang, Yuanfei Yao, Dan Su, Yue ma, Lin Fang, Yanqiao Zhang

**Affiliations:** 1 Department of Gastrointestinal Medical Oncology, Harbin Medical University Cancer Hospital, Harbin, Heilongjiang Province, China.; 2 Translational Medicine Research and Cooperation Center of Northern China, Heilongjiang Academy of Medical Sciences, Harbin, Heilongjiang Province, China.

**Keywords:** USP5, proliferation, metastasis, pancreatic cancer, deubiquitination, STAT3 signaling

## Abstract

**Purpose:** Ubiquitin specific peptidase 5 (USP5) has been reported to promote the progression of several malignant tumors. It may affect cancer development via modulating cell cycle and colony formation. In pancreatic cancer, the biological function of USP5, especially in migration and invasion remains unclear.

**Methods:** USP5 protein expression levels in primary pancreatic cancer and lymph node metastasis tissues were detected using immunohistochemistry (IHC). χ^2^ test, Kaplan-Meier analysis, univariate and multivariate analyses were used to evaluate the relationship between USP5 expression and clinicopathological feature. RT-qPCR were carried out to quantitate the mRNA expression levels of USP5 in pancreatic cancer cell lines. CCK8 and Colony formation assay were performed to prove how USP5 works in proliferation. Evaluation of tumor metastasis was made by Transwell and wound healing assay. EMT and STAT3 signaling related markers were detected by western blot.

**Results:** (1) USP5 protein expression levels were related to tumor differentiation, CEA and CA19-9 level. (2) Univariate and multivariate analyses showed that high USP5 expression is an unfavorable prognostic factor for pancreatic cancer. Kaplan-Meier analysis directly indicated that patients with high USP5 expression had shorter overall survival. (3) Increased USP5 expression is related to pancreatic cancer in both proliferation and metastasis. (4) USP5 was proved to mediate STAT3 signaling in pancreatic cancer cells.

**Conclusions:** The results suggest that USP5 is highly expressed and might have clinical significance for pancreatic cancer patients. High USP5 expression promotes both progression and metastasis by activating STAT3 signaling. Thus, USP5 might be a potential target in pancreatic cancer treatment.

## Introduction

Pancreatic cancer is an aggressive tumor with higher malignancy and higher mortality rates. Accounting for, 80% of all cases were found distant metastasis at the time of diagnosis 1, 2. Therefore, it will be meaningful to screen biomarkers and find targeted therapies for pancreatic cancer.

Ubiquitination is a major mechanism in post-translational modification of protein. Deubiquitinating enzymes (DUBs) regulate protein degradation by deubiquitinating the substrate protein 3. Accumulating evidence has confirmed that ubiquitin-specific proteases (USPs), as an important family of DUBs, have been involved in tumorigenesis and malignancy by regulating other proteins. USP5 is one of the important members of USP family. USP5 have been reported as an oncoprotein in diverse cancers including myeloma 4, non-small cell lung cancer 5, hepatocellular carcinoma 6, colorectal cancer 7, ovarian cancer 8 and pancreatic cancer 9. USP5 have been studied in the development of pancreatic cancer 9, 10. However, the role of USP5 in pancreatic cancer still needs to be studied.

In this study, we reported that USP5 is highly expressed in pancreatic cancer, especially in metastasis. The expression of USP5 is associated with poor prognosis. We also found that increased USP5 expression is related to pancreatic cancer in both proliferation and metastasis *in vitro*. USP5 was also proved to mediate STAT3 signaling in pancreatic cancer cells. This study indicated that USP5 might be a potential target for the treatment of pancreatic cancer.

## Materials and Methods

### Patients and samples

Inclusion criteria of patients was described as follows, (1) Patients underwent surgery and following treatment at Harbin Medical University Cancer Hospital from December 2003 to June 2010 with pathologically confirmed pancreatic cancer. (2) All patients signed informed consent. (3) Patients with adequate pathological tissue. Among them, 11 patients contained primary tumor tissues and its corresponding lymph node metastasis tissue samples. We excluded patients who did not sign the informed consent or with no enough tumor tissue samples. The samples were stored at -80 °C before paraffin-embedded. This study was approved by the Ethics Committee of Harbin Medical University Cancer Hospital. The clinical pathological features of patients were extracted from medical records.

### Immunohistochemical staining (IHC)

The expression of USP5 in pancreatic cancer tissues was detected using IHC. The sections were processed for immunohistochemical staining as previously reported 11. The 4-μm-thick sections were deparaffinized before heated in citrate buffer (0.01 M). Then they were incubated with 0.3% H2O2 and rehydrated. After blocking, the sections were incubated with anti-USP5 (1:100 Proteintech Wuhan Sanying, Wuhan, China) antibody at 4 °C overnight. After washing several times in phosphate buffered saline (PBS), the sections were incubated in the biotinylated secondary antibodies. After that, the slides were washed in PBS again, exposed to diaminobenzidine, and counterstained with hematoxylin. After serial dehydration, the slides were mounted under a coverslip for microscopic examination. As for blank control and negative control, the slides were incubated with PBS and omitting the primary antibody, respectively. The intensity of USP5 staining was scored as 0 (no signal), 1 (weak), 2 (moderate), and 3 (marked). Percentage score was assigned as 1 (0-25%); 2 (26-50%); 3 (51-75%); and 4 (76-100%). The scores of each tumor sample were multiplied to give a final score of 0-14. Two pathologists were asked to evaluate and record the IHC results in all cases independently, without prior knowledge of the clinical data. With a critical value of 9, we divided the expression to low expression (<9) and positive expression (≥9).

### Cell culture and transfection

Human pancreatic cancer cell lines (PL45, PSN1, MIAPaCa2, Panc1, HPAFII, panc03.27, BxPC3, Capan2, AsPC1, CFPAC1, Hs766T, SW1990, Capan1) and the normal pancreatic ductal epithelial cells (HPDE6.C7) were purchased from American Type Culture Collection. All cell lines have been authenticated recently. Cells were maintained in RPMI 1640 (HPDE6.C7 AsPC1, Panc03.27, PSN1, HPAFII), DMEM (SW1990, BxPC3, Panc1, PL45, Hs766T, MiaPaCa-2), IMDM (CFPAC1, Capan1), McCoy's 5A (Capan2) medium. All the cell lines were supplemented with 10% fetal bovine serum (FBS) and 1%PS, except for MiaPaCa-2 (10%FBS and 2.5% horse serum). They were kept at 37 °C with 5% CO2 in an incubator.

We selected Panc1 cells with low USP5 expression for USP5 gene overexpression and AsPC1 cells with high USP5 expression for USP5 gene knockdown. Both cell lines were seeded in 6-well plates at a concentration of 3×10^5^ cells/well. Both overexpression and shRNA plasmid (Vigene Biosciences, Shandong, China) was transfected using Lipofectamine 2000 transfection reagent (Thermo Fisher Scientific, USA). Empty pENTER plasmid and shRNA control were used as Negative control.

### Quantitative real-time PCR (RT-qpcr) assay

Total RNA was extracted by using the Trizol reagent (Thermo fisher Scientific, Waltham, MA, USA), and the reverse-transcription reactions were performed by using an M-MLV Reverse Transcriptase kit (Invitrogen, Carlsbad, CA, USA). To find out mRNA levels of USP5 and β-actin, RT-qPCR was performed by using a standard SYBR Green PCR kit (Toyobo Life Science, Shanghai, China), according to the manufacturer's instructions. The primers were as follows:

- USP5-F: GCTGCTGTCAGTATTACCGAC, and;
- USP5-R: AAAGCCCAGAAACGTGTTCATA.

### Cell viability assay

Panc1 and AsPC1 cells were treated with siRNAs and overexpression plasmids. The next day, cells were seeded at a density of 1000 cells per well in 96 well plates. Forty-eight hours later, 10 μL of Cell Counting Kit-8 reagent (Dojindo, Kumamoto, Japan) was added into each well, and cultured for 1 hour at 37 °C in the dark, according to the manufacturer's instruction. The absorbance of each well was measured at 450 nm every day for the next 3 days.

### Colony formation assay

Panc1 cells (1000 cells/well) and AsPC1 cells (1000 cells/well) were seeded into 6-well plates and cultured for 7 days, respectively. When the clone is visible, the plate was stained with crystal violet solution for 3 min and then washed with ddH_2_O.

### Invasion assay

As for invasion assays, Panc1 cells (1×10^5^ cells/well) and AsPC1 cells (1×10^5^ cells/well) were added into top chamber of Transwell inserts coated with 40 μL Matrigel (1:15,Corning, NY, USA). The bottom chamber was filled with 750μL of medium containing with 10% FBS, while the top chamber was filled with 500 μL of pure medium. After cultured at 37 °C for 24 hours, the chamber was washed in PBS and fixed in 4% paraformaldehyde for twenty minutes. After that, it was stained with crystal violet staining solution (Beyotime, Shanghai, China) and taken pictures under a microscope. The counting was analyzed and quantified via ImageJ (National Institutes of Health).

### Migration assay

Panc1 cells (4×10^4^ cells/well) and AsPC1 cells (3×10^4^ cells/well) with different treatments were seeded into scratch chamber. The chamber was moved the next day and taken pictures under the microscope (0 h, 24 h).

Transwell inserts without Matrigel was used as migration assay.

### Western blotting

Total proteins were harvested from cultured cells as described previously 11. Proteins were separated by 10% SDS-polyacrylamide gel electrophoresis and then transferred to PVDF membranes. 5% nonfat milk was used to block the membrane. After that, the membrane was incubated with primary antibodies against USP5 (1:1000 Proteintech Wuhan Sanying, Wuhan, China), actin (1:5000, Santa Cruz Biotechnology, lnc., Dallas, TX, U.S.A.), STAT3 (1:1000, Affinity, Changzhou, China), pSTAT3 (1:500, Affinity, Changzhou, China) and other EMT markers (N-cadherin, 1:1000, Proteintech Wuhan Sanying, Wuhan, China, vimentin, 1:1000, Arigo, Taiwan, China, β-catenin, 1:1000, Arigo, Taiwan, China), followed by horseradish peroxidase (HRP)-conjugated secondary antibodies (1:5000 Proteintech Wuhan Sanying, Wuhan, China). Immunoreactive proteins were detected using a chemiluminescence solution (Thermo fisher Scientific).

### Bioinformatics analysis

GEPIA and ICGC databases were used to validate our experimental results. GEPIA directly showed the differential expression of USP5 in pancreatic cancer and normal tissues. As for ICGC, we downloaded and re-analyzed USP5 expression and its correlation with clinical prognosis.

### Statistical analysis

All the results were presented as the mean ± standard using GraphPad prism 7.0 (GraphPad Software, USA) and SPSS statistical software 18.0 (IBM Corp., Armonk, NY, USA). Χ^2^ test was used to analyze the relationship between USP5 expression and other clinicopathological features. The Cox proportional hazard model was generated for univariate and multivariate regression analysis to identify prognostic factors. Kaplan-Meier survival curves were constructed using a logrank test. Pearson correlation coefficient analysis was conducted to test for correlation between two quantitative measurements. One-way analysis of variance (ANOVA) and Tukey's posttest were used to compare multiple groups of data. *P*-value < 0.05 was considered statistically significant; for all results * denotes *P* < 0.05; ** *P* < 0.01 and *** *P* < 0.001. Each experiment was performed with at least three independent experiments.

## Results

### Association between USP5 and the clinicopathological features in pancreatic cancer patients

To identify the regulatory roles of USP5, USP5 protein levels were tested in 94 pancreatic cancer patients by IHC. Among the samples, 61 patients had high expression of USP5 in tumor tissues (details shown in Table 1). The results showed that USP5 expression level is related to tumor differentiation, CEA and CA19-9 levels (*P<* 0.05).

### High USP5 expression level is an independent prognostic factor in pancreatic cancer

**Figure 1:**
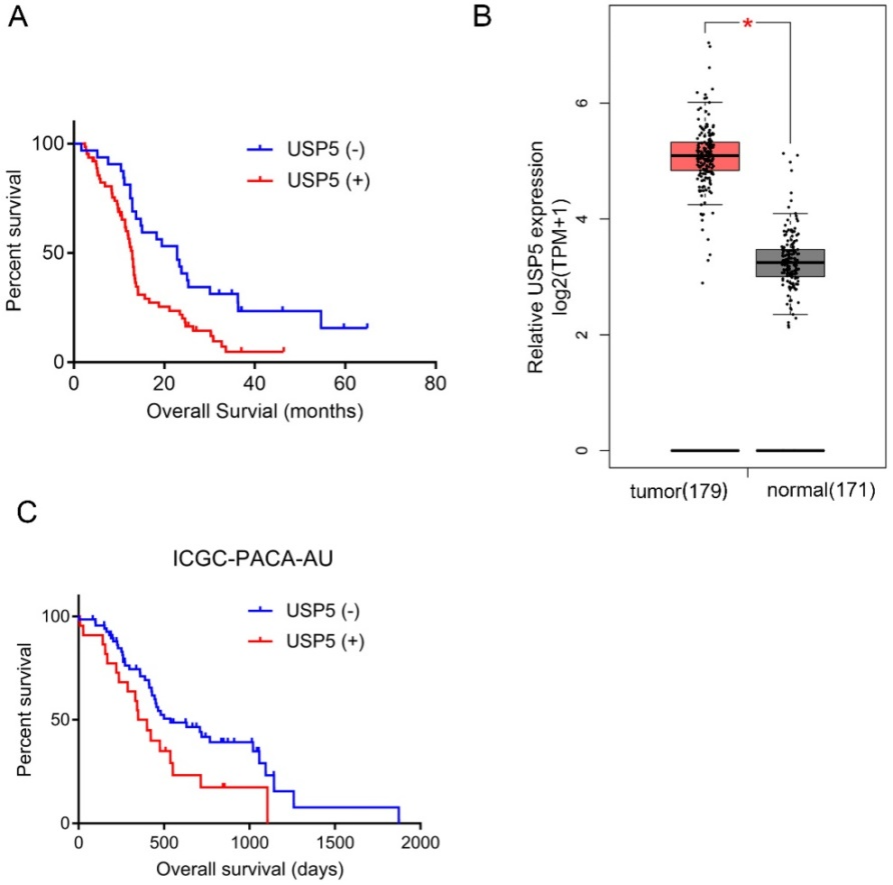
** High USP5 expression suggests poor prognosis and shorter OS.** (A) The overall survival curves for the high-USP5 expression group and the low-USP5 expression group; the difference is statistically significant (*P* < 0.05). (B) USP5 expression levels in normal or pancreatic cancer tissues retrieved from GEPIA database. TPM, transcripts Per Million. (C) The overall survival curves for USP5 in ICGC database (ICGC-PACA-AU), *P*< 0.05.

As shown in Table 2, univariate analysis found that USP5 expression (*P*=0.001), tumor differentiation (*P<* 0.001), CEA level (*P<* 0.05) and CA19-9 level (*P<* 0.01) were significant prognostic predictors in pancreatic cancer. Multivariate analysis identified that USP5 expression (*P*< 0.05) and differentiation (*P*< 0.05) were independent prognostic factors. Kaplan-Meier survival analysis was performed to explore the association between USP5 expression and survival time in pancreatic cancer patients (Fig. 1A). We found that the median OS of patients with positive USP5 expression (12.9 months) was remarkably shorter than patients with negative USP5 expression (22.8 months). In general, the results indicated that patients with negative USP5 expression have a longer survival time than those with positive USP5 expression in pancreatic cancer. In the public GEPIA database, we also found that USP5 expression was significantly higher in pancreatic cancer tissue than in normal tissue (Fig. 1B). Additionally, we verified Kaplan-Meier survival analysis in ICGC Data Portal (ICGC-PACA-AU), which is consistent with our result (Fig. 1C).

### Increased USP5 expression suggests correlation with pancreatic cancer metastasis

**Figure 2:**
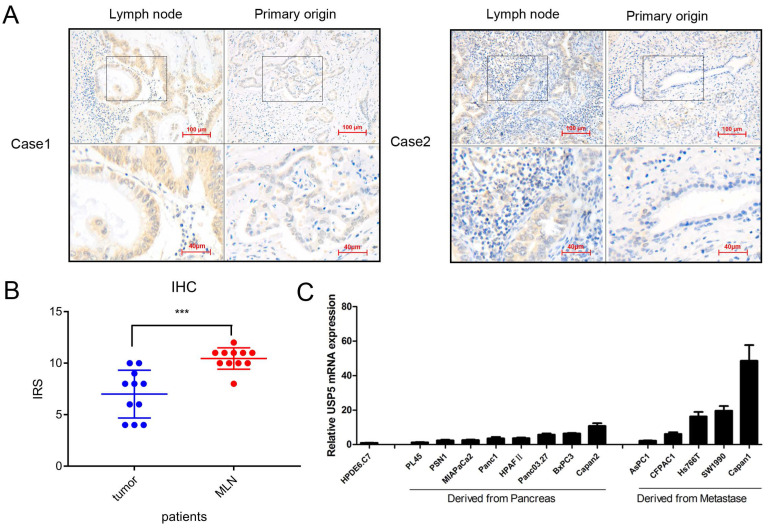
** Increased USP5 expression suggests correlation with pancreatic cancer metastasis.** (A) USP5 expression in lymph node and primary origin in paraffin-embedded tissues from pancreatic cancer patients.(magnification, 200×) (B) IHC score in metastasis lymph node (MLN) and primary origin. (C) The expression of USP5 examined by qPCR in eight cell lines derived from pancreatic cancer, five cell lines derived from metastasis and one normal pancreatic epithelial cell line. *P < 0.05; **P < 0.01; ***P < 0.001.

We detected USP5 expression in 11 pairs of primary pancreatic tumors and lymph node metastasis tissues (Fig. 2A). Interestingly, the results showed that the expression of USP5 was elevated in patients with lymph node metastasis (Fig. 2B). The USP5 mRNA level was detected in normal pancreatic cell and multiple pancreatic cancer cells, showing that USP5 mRNA expression was significantly increased in metastasis cells than primary cancer cells (Fig. 2C). We also confirmed that USP5 mRNA was highly expressed in pancreatic cancer cell lines compared with normal pancreatic cells. Taken together, we speculated that increased USP5 level is associated with tumor metastasis.

### USP5 overexpression promotes the progression and metastasis of pancreatic cancer

**Figure 3:**
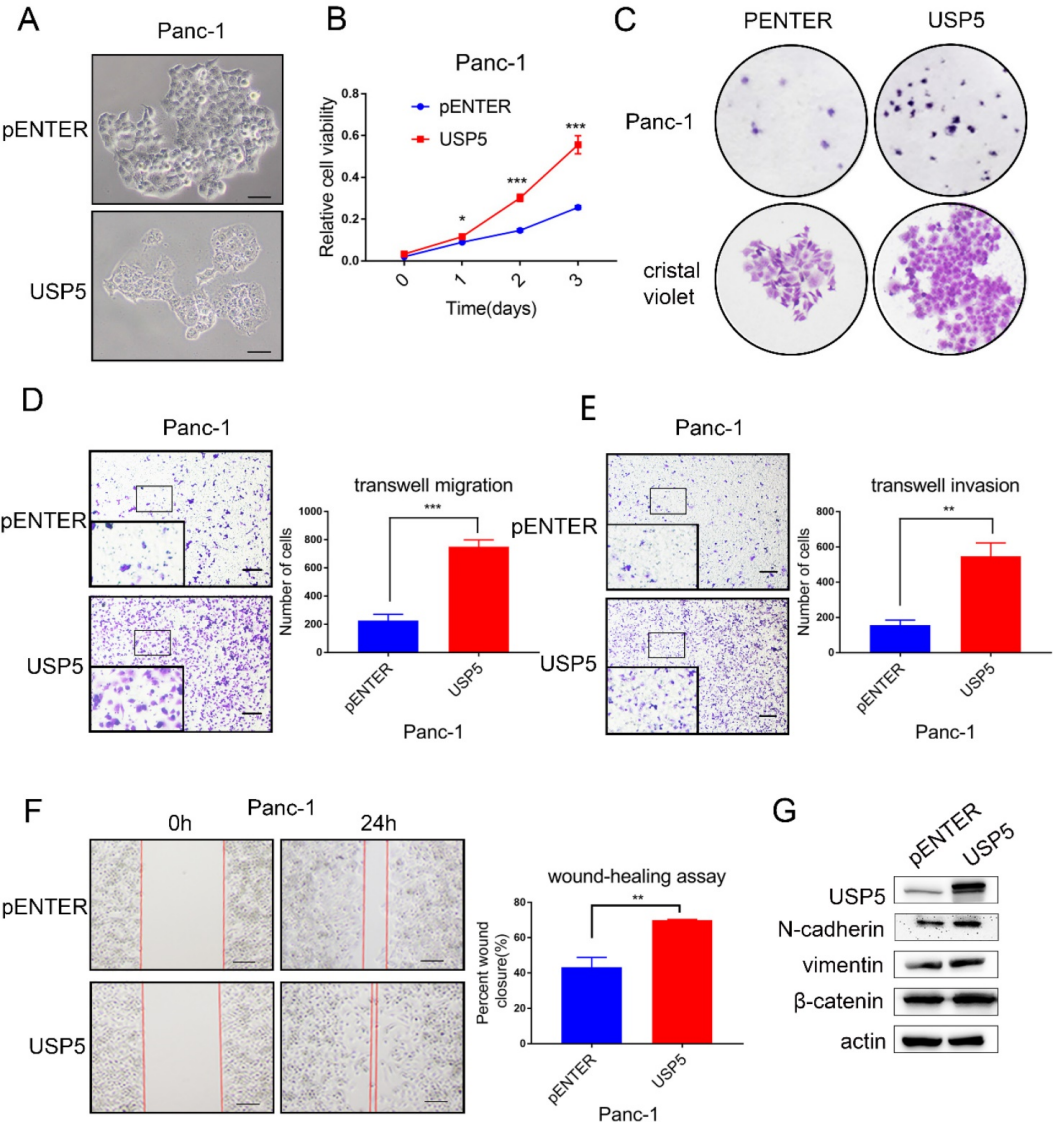
** USP5 overexpression promotes the progression and metastasis of pancreatic cancer.** (A) Overexpressed USP5 in Panc1 cells.(B) Overexpression of USP5 improved the viability of Panc1 cells. (C) Overexpression of USP5 enhanced colony formation of the Panc1 cells. (D-E) Transwell assays evaluated the effect of USP5 overexpression on migration and invasion of Panc1 cells after 24 hours. (F) Wound healing assay of Panc1 cells transfected with pENTER and USP5. Results were measured after 24 hours. (G) The expression level of EMT related markers in USP5 overexpression cells. Scale bar 100 µm *P<0.05, **P<0.01, ***P < 0.001, Student's unpaired t-test.

To further evaluate the biological function of USP5 *in vitro* experiments, we transfected USP5 overexpressing plasmid and observe cell morphology under microscope (Fig. 3A). CCK-8 assay and colony formation experiments showed that overexpression of USP5 in Panc1 cells dramatically increased the proliferation (Fig. 3B-C). Transwell assay and wound healing assay were used to assess the effects of USP5 in metastasis. Transwell assay demonstrated that overexpress USP5 promote both migration and invasion after 24 hours (Fig. 3D-E). Similarly, wound healing assay showed that the healing capability of USP5 overexpression was significantly increased compared with negative control cells after the observation of 24 hours (Fig. 3F). Western blot analysis further affirmed that the expression level of EMT related markers (N-cadherin, vimentin and β-catenin) have been changed relatively (Fig. 3G).

### USP5 knockdown inhibits the progression and metastasis of pancreatic cancer

**Figure 4:**
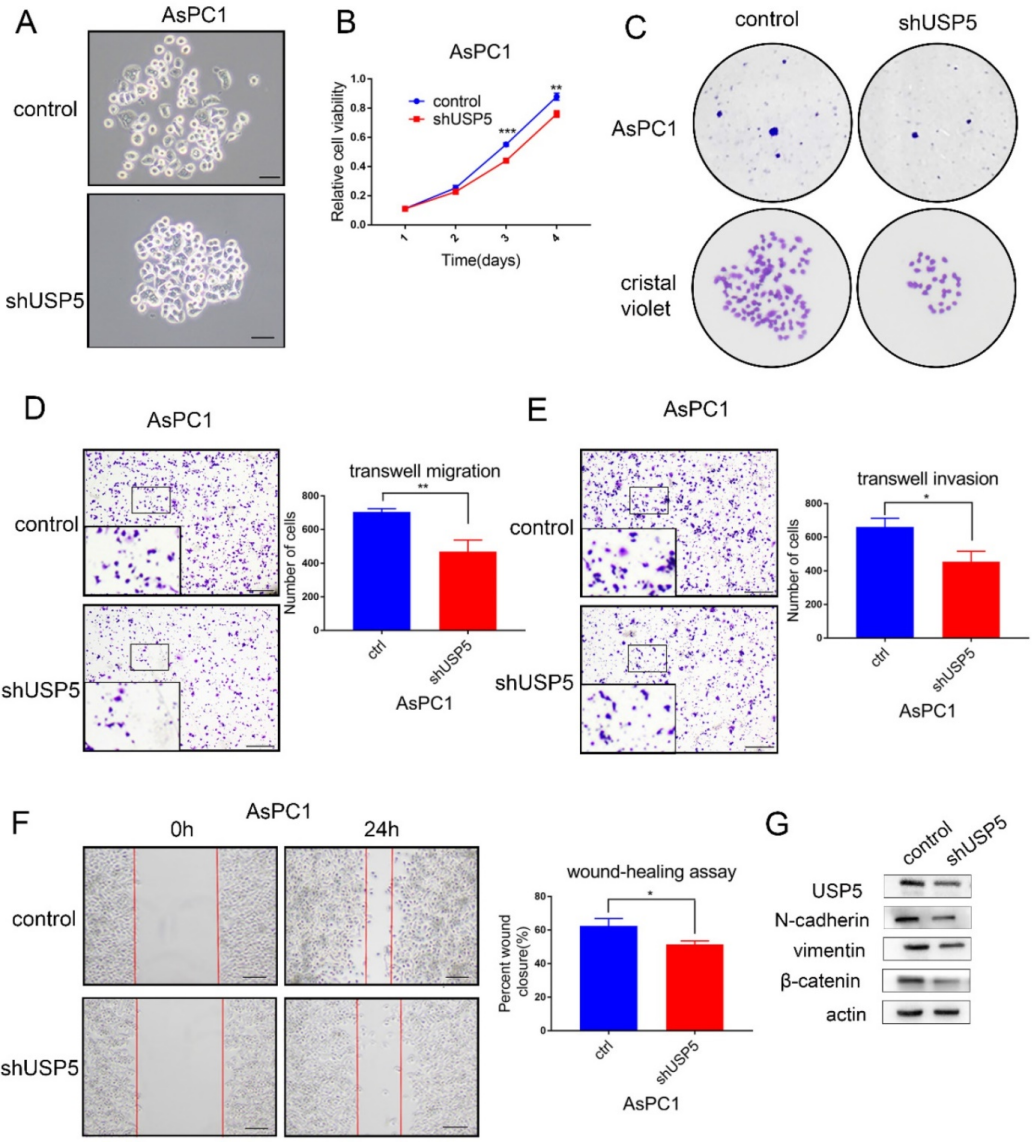
** USP5 knockdown inhibits the progression and metastasis of pancreatic cancer.** (A) Knockdown USP5 in AsPC1 cells. (B) Knockdown USP5 inhibit the viability of AsPC1 cells.(C) Knockdown USP5 inhibit colony formation of the AsPC1 cells. (D-E) Transwell assays evaluated the effect of USP5 knockdown on migration and invasion in AsPC1 cells after 24 hours. (F) Wound healing assay of AsPC1 cells transfected with shUSP5. Results were measured after 24 hours. (G) The expression level of EMT related markers in USP5 knockdown cells. Scale bar 100μm *P<0.05, **P<0.01, ***P< 0.001, Student's unpaired t-test.

To further identify the role of USP5 in pancreatic cancer, we knockdown the expression of USP5 in AsPC1 cells by shRNA. Cell morphology was observed under microscope (Fig. 4A). The cell viability assay and colony formation assay revealed that cell proliferation was significantly inhibited in USP5 knockdown cells (Fig. 4B-C). In contrast with USP5 overexpression, knockdown USP5 inhibited cell invasion and migration (Fig. 4D-G). Taken together, these results suggested that USP5 could evidently promote pancreatic cancer cells proliferation and metastasis.

### USP5 mediates STAT3 signaling in pancreatic cancer

**Figure 5:**
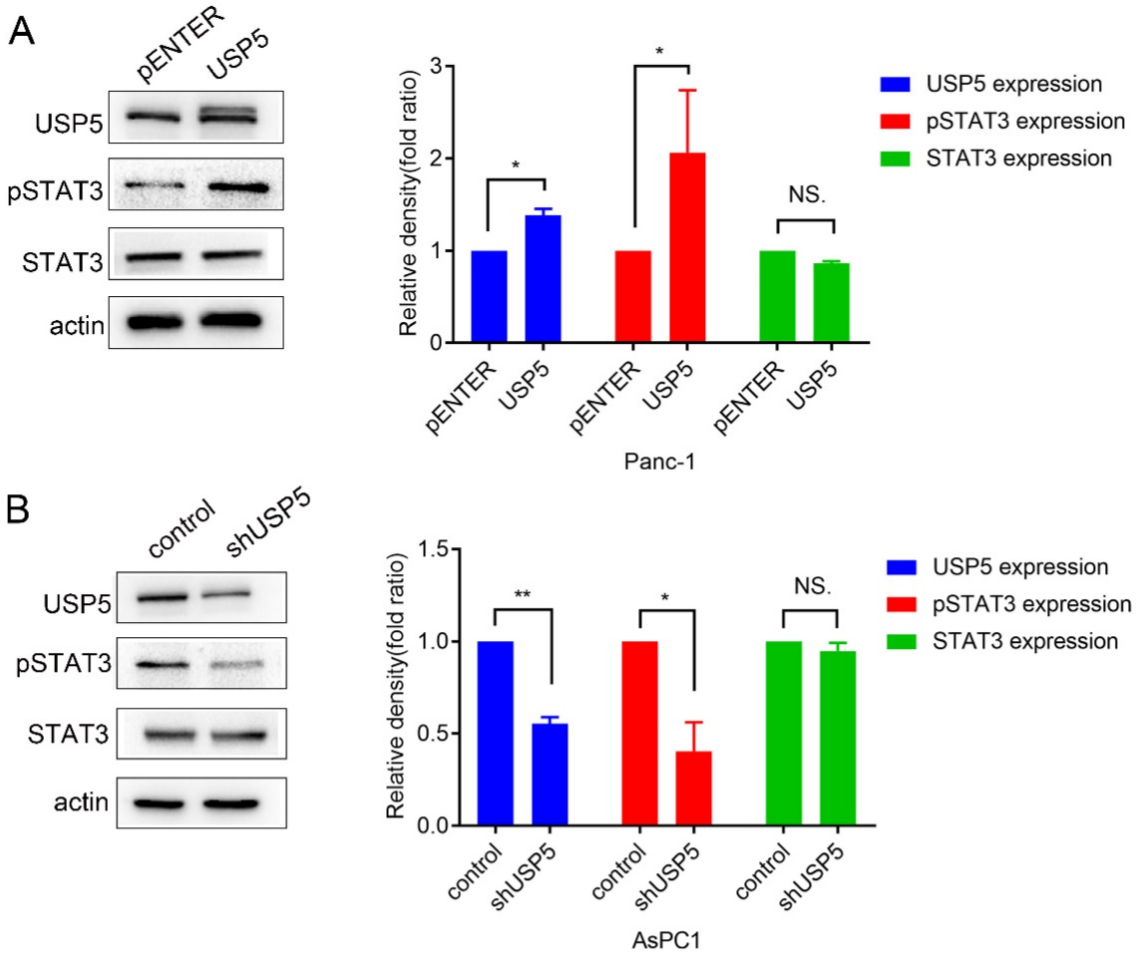
** USP5 was proved to mediate STAT3 signaling in pancreatic cancer cells.** (A) Overexpress USP5 in Panc1 cells activate STAT3 signaling. (B) Knockdown USP5 in AsPC1 cells inhibit STAT3 signaling. Data were quantified by densitometric analysis with ImageJ software. *P < 0.05; **P < 0.01; ***P < 0.001.

STAT3 is often overexpressed or overactivated in several tumors 12. A recent study also reported that the ubiquitin ligase Fbw7 induced STAT3 ubiquitination for degradation in diffuse large B-cell lymphoma 13. However, the effect of USP5 in STAT3 signaling was still unclear. In this study, we found that STAT3 was overactivated, along with the upregulation of USP5 (Fig. 5A). In contrast, knockdown of USP5 suppressed the activation of STAT3 (Fig. 5B).

## Discussion

Ubiquitin-proteasome pathway is a common pathway for protein degradation. Increasing evidence has shown that USP family has important significance in tumor cell apoptosis 4, cycle 10, metastasis 6, drug resistance 14 and other biological activities 15, 16. For instance, USP4 promote tumor progression by regulating β-Catenin Signaling 17. It has been proven that USP2 promotes breast cancer metastasis by deubiquitinating MMP2 18. Also, USP22 is responsible for hepatocellular carcinoma migration 19. USP5 has been reported in targeting several proteins, such as P53, FOXM1, SLUG, βcatenin, c-Maf, TUFM, HDAC2 et al 4-9, 20, 21. USP5 has also been implicated in a wide variety of cellular events, such as the efficient repair of DNA Double-Strand Breaks, alternative RNA Splicing, neural pain, tumor cell proliferation and progression 6, 9, 15, 16, 22. Intriguingly, USP5 has been confirmed to mediate p53 signaling 20, wnt-β-catenin pathway 5, Type I interferon signaling pathway 23, Notch and RTK signaling pathway 24. A recent study confirmed that upregulated USP5 can promote pancreatic cancer 10. However, its role in pancreatic cancer still remains further studied.

The expression status and clinical significance of USP5 in pancreatic cancer has not been reported extensively. In the current study, we discovered that USP5 expression level is related to tumor differentiation, CEA and CA19-9 levels. Univariate and multivariate analyses demonstrated that high USP5 expression is an independent risk factor for pancreatic cancer. Our results also showed that differentiation is an unfavorable independent risk factor for pancreatic cancer, which have been proved by previous study 25. In short, patients with high USP5 expression tend to have a poor prognosis, which is consistent with publicly databases.

Recently, there have been several reports on how USP5 promote the growth of cancer. By stabilizing Tu translation elongation factor, USP5 could regulate colorectal cancer cell growth 7. Moreover, USP5 stabilized FoxM1 protein in pancreatic ductal adenocarcinoma and regulated cell proliferation via FoxM1 proteins 9. This has been confirmed in our experiments.

In previous study, USP5 was found promoting EMT by stabilizing SLUG in hepatocellular carcinoma 6. In our study, we reported that the expression of USP5 is higher in metastasis than in primary tumor. This was confirmed in clinical patient samples and pancreatic cancer cell lines, respectively. To further investigate how USP5 affect pancreatic cancer migration and invasion, we overexpressed USP5 in Panc1 cells and knockdown USP5 in AsPC1 cells. Results elucidated that USP5 contribute to cell invasion and metastasis in pancreatic cancer.

The effect of USP5 in progression and migration in pancreatic cancer has been confirmed, but its mechanism was still unknown. In this study, we confirmed that USP5 mediated STAT3 signaling. The signal transducer and activator of transcription 3 (STAT3) is an important signaling mediator, which plays significant roles in a vast array of biological processes 26-28. It has been confirmed that overexpression or overactivation of STAT3 is required for tumorigenesis and metastasis. Thus, USP5 may promote pancreatic cancer progression and metastasis through enhancing STAT3 signaling.

Small molecules inhibitor WP1130 29 and Chinese medicine Formononetin 6 were reported to inhibit USP5, which further indicated that targeting USP5 might be an effective strategy in pancreatic cancer treatment.

In conclusion, our study identifies that USP5 is highly expressed in pancreatic cancer and might have clinical significance for patients with pancreatic cancer. USP5 overexpression is critical in both pancreatic cancer progression and metastasis via enhancing STAT3 signaling. Therefore, USP5 might be a potential novel target gene in pancreatic cancer.

## Figures and Tables

**Table 1:** Correlation between USP5 expression and clinicopathological features of patients with pancreatic cancer

Clinicopathological feature	USP5 expression		*P* value
	Low	High	
**Age (years)**			0.8877
≤50	8	14	
>50	25	47	
**Sex**			0.3789
Male	22	35	
Female	11	26	
**Tumor site**			0.6346
Pancreatic head	19	32	
Pancreatic body and tail	14	29	
**Differentiation**			**0.02359**
High differentiation	10	7	
Middle and low differentiation	23	54	
**T stage**			0.3868
T1.2	9	22	
T3.4	24	39	
**N stage**			0.9941
N0	30	54	
N1	3	7	
**M stage**			1.0000
M0	32	59	
M1	1	2	
**AJCC (pTNM)**			0.9508
Ⅰ	9	17	
ⅡⅢⅣ	24	44	
**CEA**			**0.0324**
Normal	28	39	
Abnormal	5	22	
**CA199**			**0.0133**
Normal	13	10	
Abnormal	20	51	

**Table 2:** Univariate and multivariate analysis of clinicopathological factors in patients with pancreatic cancer

Variable	Univariate analysis		Multivariate analysis			
	HR (95%CI)	*P* value	HR (95%CI)			*P* value
Sex	0.71 (0.449-1.133)	0.152				
Age	1.61 (0.928-2.805)	0.898				
USP5	2.28 (1.392-3.734)	**0.001**	1.72 (1.014-2.90)			**0.044**
Tumor site	0.69 (0.440-1.089)	0.112				
Differentiation	0.29 (0.148-0.561)	**<0.001**	2.44 (1.200-4.940)			**0.014**
T stage	1.12 (0.708-1.773)	0.627				
N stage	1.11 (0.554-2.229)	0.765				
M stage	2.42 (0.750-7.799)	0.139				
pTNM	0.94 (0.580-1.509)	0.784				
CEA	060 (0.373-0.952)	**0.030**	0.91 (0.543-1.534)			0.729
CA199	0.43 (0.248-0.753)	**0.003**	0.58 (0.315-1.075)			0.084

## Abbreviations

USP5: Ubiquitin specific peptidase 5
IHC: immunohistochemistry
DUBs: Deubiquitinating enzymes
USPs: Ubiquitin-specific proteases
STAT3: signal transducer and activator of transcription 3
qPCR: Quantitative real-time PCR
MLN: metastasis lymph node
